# The XCL1-Mediated DNA Vaccine Targeting Type 1 Conventional Dendritic Cells Combined with Gemcitabine and Anti-PD1 Antibody Induces Potent Antitumor Immunity in a Mouse Lung Cancer Model

**DOI:** 10.3390/ijms25031880

**Published:** 2024-02-04

**Authors:** Ke Zhang, Qimuge Wuri, Zongyu Cai, Xueli Qu, Shiqi Zhang, Hui Wu, Jiaxin Wu, Chu Wang, Xianghui Yu, Wei Kong, Haihong Zhang

**Affiliations:** 1 National Engineering Laboratory for AIDS Vaccine, School of Life Science, Jilin University, Changchun 130012, China; zke20@mails.jlu.edu.cn (K.Z.); wrqmg21@mails.jlu.edu.cn (Q.W.); caizy22@mails.jlu.edu.cn (Z.C.); xlqu21@mails.jlu.edu.cn (X.Q.); sqzhang21@mails.jlu.edu.cn (S.Z.); topwuhui@jlu.edu.cn (H.W.); wujiaxin@jlu.edu.cn (J.W.); wangchu18@jlu.edu.cn (C.W.); xianghui@jlu.edu.cn (X.Y.); weikong@jlu.edu.cn (W.K.); 2 Key Laboratory for Molecular Enzymology and Engineering, The Ministry of Education, School of Life Sciences, Jilin University, Changchun 130012, China

**Keywords:** immunotherapy, DNA vaccine, cDC1, XCR1, XCL1, gemcitabine, anti-PD1 antibody

## Abstract

With the advent of cancer immunotherapy, there is a growing interest in vaccine development as a means to activate the cellular immune system against cancer. Despite the promise of DNA vaccines in this regard, their effectiveness is hindered by poor immunogenicity, leading to modest therapeutic outcomes across various cancers. The role of Type 1 conventional dendritic cells (cDC1), capable of cross-presenting vaccine antigens to activate CD8^+^T cells, emerges as crucial for the antitumor function of DNA vaccines. To address the limitations of DNA vaccines, a promising approach involves targeting antigens to cDC1 through the fusion of XCL1, a ligand specific to the receptor XCR1 on the surface of cDC1. Here, female *C57BL/6* mice were selected for tumor inoculation and immunotherapy. Additionally, recognizing the complexity of cancer, this study explored the use of combination therapies, particularly the combination of cDC1-targeted DNA vaccine with the chemotherapy drug Gemcitabine (Gem) and the anti-PD1 antibody in a mouse lung cancer model. The study’s findings indicate that fusion antigens with XCL1 effectively enhance both the immunogenicity and antitumor effects of DNA vaccines. Moreover, the combination of the cDC1-targeted DNA vaccine with Gemcitabine and anti-PD1 antibody in the mouse lung cancer model demonstrates an improved antitumor effect, leading to the prolonged survival of mice. In conclusion, this research provides important support for the clinical investigation of cDC1-targeting DNA vaccines in combination with other therapies.

## 1. Introduction

Cancer immunotherapy has witnessed significant strides in the past two decades [1,2]. As the field continues to evolve, it has become evident that CD8^+^ cytotoxic T lymphocytes (CTLs) play a pivotal role in tumor immunity [3]. Capitalizing on their capacity to induce tumor-specific T cell production and activation, cancer vaccines have emerged as an enticing strategy to modulate tumor growth dynamics [4,5]. While DNA vaccines have garnered widespread attention owing to their simplicity, safety, and proficiency in inducing specific immune responses, their clinical utility is hindered by limitations, notably weak immunogenicity [6]. For the immune response induced by DNA vaccines, antigen-presenting cell (APC) uptake, processing, and especially the presentation of vaccine antigens to CD8^+^T cells are key to this process [6,7]. Therefore, in order to enhance the immunogenicity and therapeutic efficacy of DNA vaccines, improving the antigen presentation of DNA vaccines is a promising strategy.

Dendritic cells (DCs), the most effective APCs, have the unique function of activating naïve T cells [8,9]. However, accumulated experimental evidence in the past suggests that there are several different DC subpopulations during the development of DCs, which are used to initiate different types of effector T cells [10,11]. Type 1 conventional dendritic cells (cDC1) have the ability to cross-present exogenous antigens to the naïve CD8^+^T cells and are key cells that initiate the cytotoxic effect T cell response [12,13,14]. Many studies have also demonstrated that cDC1 are crucial for antitumor immunity [12,15], and in IRF8^−^/^−^ or Batf3^−^/^−^ mice with cDC1 deficiency, CD8^+^T cell responses are severely affected, leading to a decrease in, or even loss of, antitumor immunity [16,17,18]. Consequently, targeting antigens to cDC1 emerges as a promising strategy to enhance the antitumor immune response. Studies have shown that cDC1 selectively express the chemokine receptor XCR1 [15,19,20,21], and in humans, XCR1 is also specificity expressed on a subpopulation of DCs (CD141^+^DCs) that are functionally homologous to mice [21,22,23,24]. Leveraging this insight, delivering antigens to cDC1 via XCL1, the ligand of XCR1, presents a promising avenue for vaccine design. This strategy finds validation in several vaccine designs, including those for COVID-19 and influenza [25,26] as well as tumor vaccines [27,28,29].

MUC1 and survivin are two ideal tumor-associated antigens (TAAs), various vaccines targeting them have been developed [30,31]. Our previous studies have shown that the combination of MUC1 and survivin antigens induces stronger antitumor effects in multiple tumor models than vaccines with the antigen alone [32,33,34]. Here, we constructed a novel DNA vaccine (XCL1-MS) that expresses XCL1, MUC1 and survivin fusion antigens to target cDC1, aiming to induce a robust CD8^+^T cell response to inhibit tumor development. However, malignancy is a complex disease that is difficult to cure with a single therapy. The combination of multiple therapies, such as vaccine and chemical drugs, as well as immune checkpoint blockades (ICBs), may be an effective strategy [35]. Therefore, we hypothesized that the combination of Gem and anti-PD1 antibody could further improve the antitumor effect of DNA vaccines in mouse lung cancer models. Our experimental findings substantiate this hypothesis, revealing that the triple therapy significantly prolongs the survival time of mice, underscoring its potential as a formidable approach in combating cancer.

## 2. Results

### 2.1. The Construction and Validation of DNA Vaccines

**Figure 1:**
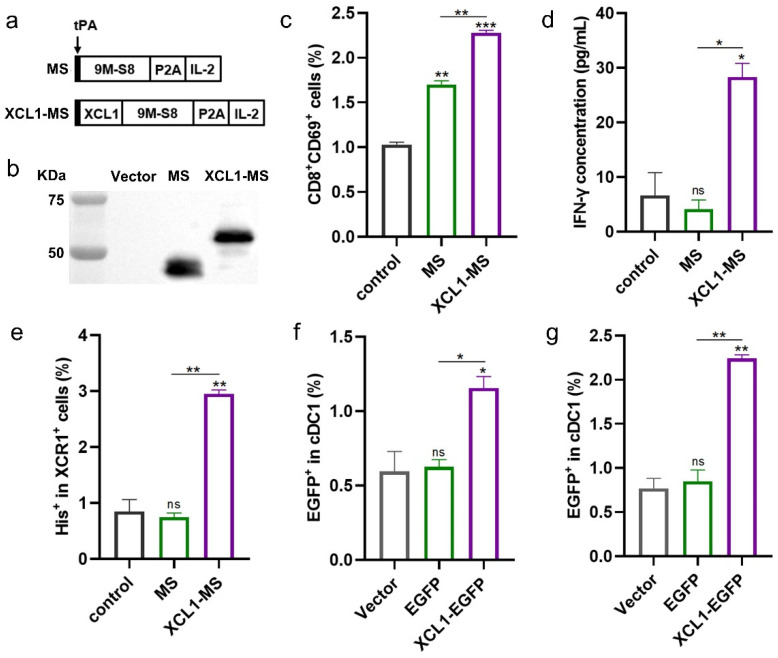
Construction and expression of two DNA vaccines. (**a**) Schematic diagrams of the two DNA plasmids used in this study (9M: 9 *MUC1 VNTRs*, S8: truncated *survivin*). (**b**) 293T cells were transfected with plasmids, and the expression of the fusion protein was verified by Western blotting. (**c**) The percentage of CD8^+^T cell activation stimulated by co-incubation of cDC1-loaded protein. (**d**) The concentration of IFN-γ secreted by activated CD8^+^T cells as described in (**c**) was detected by ELISA. (**e**) The binding between the protein and the XCR1 receptor was detected by the protein’s His tag. (**f**) cDC1 targeting was detected in lymph nodes of mice immunized after 3 days. (**g**) cDC1 targeting was detected in lymph nodes of mice immunized after 6 days. (**b**,**e**,**g**) were performed twice with similar trends. (**c**,**d**) show two wells from one experiment. * *p* < 0.05, ** *p* < 0.01, *** *p* < 0.001, and ns: no significance.

To test whether additional targeting using XCL1 helps induce a stronger immune response, the DNA vaccine XCL1-MS was constructed containing the *XCL1*, *MUC1*, and *survivin* and *IL-2* adjuvant fusion genes (Figure 1a). The expression of fusion proteins encoded by DNA vaccines was detected by Western blotting using anti-survivin antibody (Figure 1b).

To determine whether XCL1 can promote the activation of CD8^+^T cells, we performed in vitro validation. In short, CD8^+^T cells were stimulated by cDC1-loaded purified MS and XCL1-MS proteins. The results showed that CD8^+^T cells were activated by both MS and XCL1-MS protein compared with those of the control (no protein added), and the activation ability of the XCL1-MS protein was significantly higher than that of the MS protein (Figure 1c and Figure S1a,b). Moreover, by detecting the IFN-γ concentration in the cell culture supernatant, it was found that only the XCL1-MS protein has the ability to stimulate the secretion of IFN-γ by CD8^+^T cells (Figure 1d). This evidence preliminarily proves that the fusion expression of XCL1 can better bind cDC1, thus enhancing the activation of CD8^+^T cells.

We further verified the binding of the chemokine XCL1 to cDC1 by receptor-binding experiments and found that the binding of XCL1-MS to the XCR1 receptor was significantly higher than that of the control (no protein added) and MS (Figure 1e and Figure S1c). To confirm the targeting ability of our vaccine to cDC1 in vivo, plasmids expressing EGFP and XCL1-EGFP were constructed. And 100 μg of EGFP or XCL1-EGFP plasmids was intramuscularly injected into the hind leg muscles of mice, just like DNA vaccines. Moreover, the lymph nodes of mice were examined at 3 and 6 days after the last immunization, and it was found that XCL1-EGFP-immunized mice had significantly higher levels of the protein detected on lymph node cDC1 than the vector and EGFP groups (Figure 1f,g and Figure S1d,e). In summary, we found that the fusion of the XCL1 improved the targeting of the vaccine against cDC1 and thus enhanced the activation of CD8^+^T cells.

### 2.2. The Immunogenicity of DNA Vaccines

**Figure 2:**
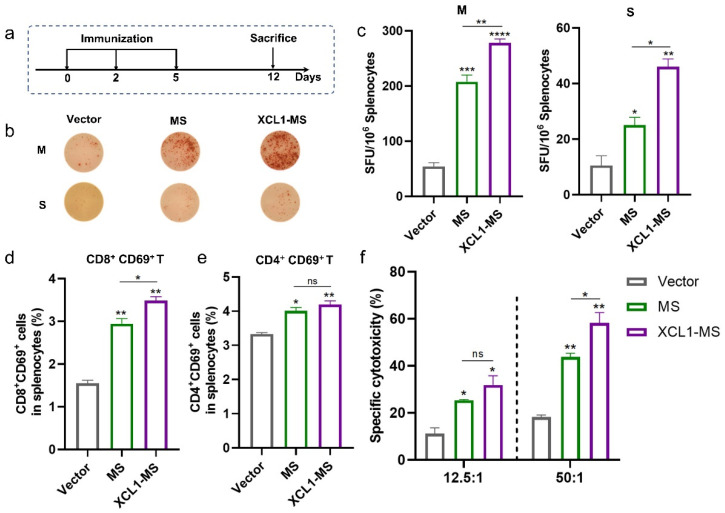
Immunogenicity analysis of DNA vaccines in *C57BL/6* mice. (**a**) Schematic of the vaccination regimen; the mice (*n* = 5) were euthanized 7 days after the last immunization. (**b**,**c**) Representative images of ELISpot and quantification of ELISpot SFU in different immune groups after stimulation (M: MUC1 protein; S, survivin protein). (**d**) Activation of CD8^+^T cells in splenocytes. (**e**) Activation of CD4^+^T cells in splenocytes. (**f**) CTL assay. Target cells labeled with CFSE were incubated with effector cells (E:T = 12.5:1 or 50:1). * *p* < 0.05, ** *p* < 0.01, *** *p* < 0.001, **** *p* < 0.0001, and ns: no significance.

To compare the immunogenicity of two DNA vaccines in mice, *C57BL/6* mice were immunized according to the schedule shown in Figure 2a. The frequency of T cells secreting antigen-specific IFN-γ was measured by ELISpot (Figure 2b,c). Both MS and XCL1-MS immunization induced a stronger antigen-specific T cell immune response than the vector, and the intensity of the cellular immunity of XCL1-MS was significantly higher than that of MS. In addition, the XCL1-MS significantly induced more CD8^+^T cell activation (Figure 2d), but the activation of CD4^+^T cells was not improved (Figure 2e). In a CTL assay, when the E:T ratio was 12.5:1, the specific killing activity of XCL1-MS was slightly higher than that of MS but with no significant difference, while when the E:T ratio was 50:1, the specific killing activity of XCL1-MS was significantly higher than that of MS (Figure 2f). These results suggest that XCL1-MS can induce a stronger specific immune response in *C57BL/6* mice.

### 2.3. The Antitumor Effect of DNA Vaccines in a Mouse Lung Cancer Model

**Figure 3:**
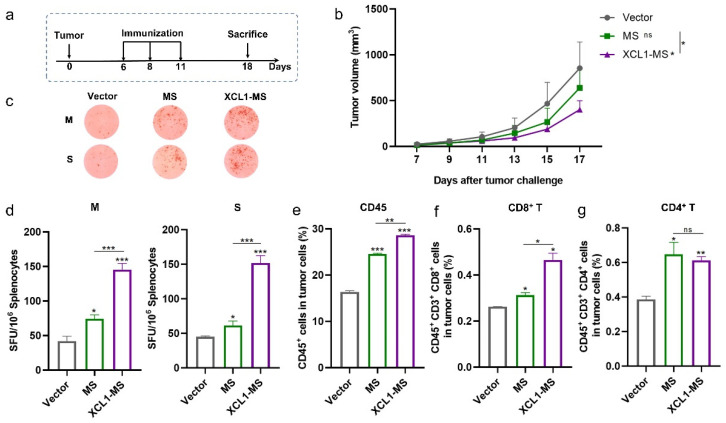
Evaluation of the antitumor effect of DNA vaccines. (**a**) Schematic diagram of the DNA vaccine treatment regimen; 5 × 10^5^ MS_f_^+^Lewis cells were injected subcutaneously into *C57 BL/6* mice on day 0, and immunization began after 6 days. (**b**) Tumor growth curves were measured every two days (*n* = 5). (**c**,**d**) Representative images of ELISpot and quantification of ELISpot SFU in different immune groups after stimulation (M: MUC1 protein; S, survivin protein). (**e**–**g**) The proportion of infiltrating CD45 (**e**), CD8^+^T cells (**f**), and CD4^+^T cells (**g**) in the tumor was measured by flow cytometry. * *p* < 0.05, ** *p* < 0.01, *** *p* < 0.001, and ns: no significance.

Next, we evaluated the therapeutic effect of the XCL1-MS vaccine using a previously laboratory-established lung cancer model. *C57BL/6* mice were subcutaneously inoculated with 5 × 10^5^ MS_f_^+^Lewis cells on day 0 and received treatment on days 6, 8, and 11 (Figure 3a). Tumor growth was monitored; the results showed that tumor growth was inhibited to some extent by both MS and XCL1-MS vaccines compared with the vector, and the inhibitory effect of XCL1-MS was significantly stronger (Figure 3b). The ELISpot assay showed that when stimulated with MUC1 or survivin, the number of T cells secreting IFN-γ was significantly increased in the MS and XCL1-MS groups compared to the vector group; moreover, the XCL1-MS group was significantly higher (Figure 3c,d). Next, we analyzed the immune cells infiltrating into the tumor. Both vaccines were able to induce more immune cells infiltrating into the tumors (Figure 3e and Figure S2), as well as more CD8^+^T and CD4^+^T cell infiltration (Figure 3f,g and Figure S2). However, compared to MS, XCL1-MS could induce more CD8^+^T cell infiltration, with no significant difference in CD4^+^T cells. A comparison of XCL1-MS with MS indicates that the fusion of XCL1 with immunogen MUC1 and survivin induced stronger immunogenicity and better antitumor activity.

### 2.4. The Antitumor Effect of the DNA Vaccine (XCL1-MS) Combined with Gem

**Figure 4:**
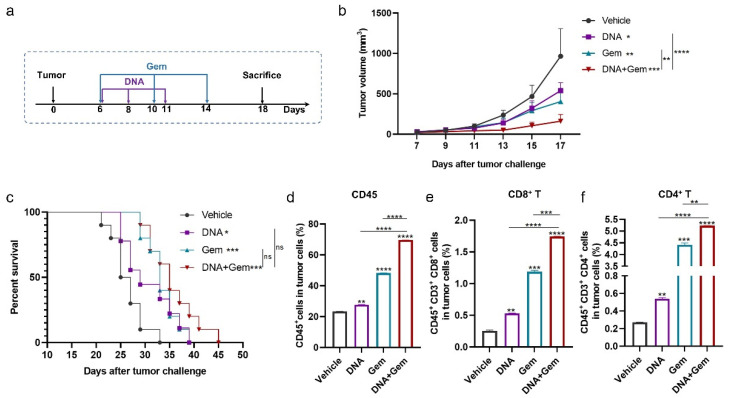
Antitumor effect of DNA vaccine (XCL1-MS) and Gem combined therapy. (**a**) Schematic diagram of treatment plan. All treatments began on day 6 after being challenged with tumor cells. (**b**) Tumor growth curves were measured every two days (*n* = 5–6). (**c**) Survival curves of mice in different treatment groups, 9–10 mice in each group. (**d**–**f**) The proportion of infiltrating CD45 (**d**), CD8^+^T cells (**e**), and CD4^+^T cells (**f**) in the tumor was measured by flow cytometry. * *p* < 0.05, ** *p* < 0.01, *** *p* < 0.001, **** *p* < 0.0001, and ns: no significance.

Gem has been widely used as an anticancer drug for the treatment of various cancers, including lung cancer [36,37,38]. Therefore, we hypothesize that the antitumor effect of DNA vaccines can be further enhanced by combining them with Gem. Mice were challenged with MS_f_^+^Lewis cells, and the treatment strategy is shown in Figure 4a. Gem administration did not affect the body weight of the mice (Figure S3a). Both treatments alone significantly inhibited tumor growth, and as expected, the combination treatment significantly enhanced the inhibition of tumor growth compared to the single treatment (Figure 4b). In the mouse survival experiment, the survival time of the mice was prolonged by both the combination and single-treatment group, compared with the vehicle. The survival time of mice in the combination group was longer than that in the single-treatment group, but there was no significant difference (Figure 4c).

Next, we analyzed the immune cells infiltrating into the tumor. The addition of Gem increased the number of T cells, and the number of CD8^+^T cells and CD4^+^T cells in the combination group was significantly higher than that in the single-treatment group (Figure 4e,f and Figure S3b,c). Therefore, the combination of DNA vaccine and Gem (dual therapy) produces better antitumor effects than single therapy.

### 2.5. The Effect of Dual Therapy on the Tumor Immune Microenvironment

**Figure 5:**
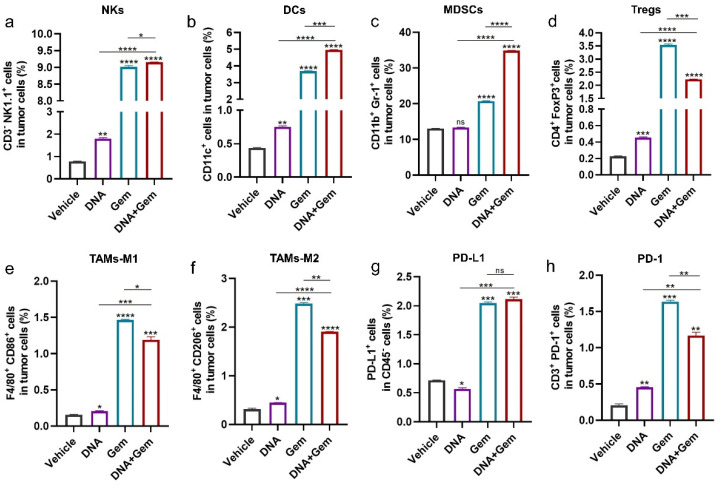
Regulation of tumor microenvironment by dual therapy. (**a**–**d**) The percentage of NKs, DCs, MDSCs, and Tregs in the tumor was analyzed by flow cytometry. (**e**,**f**) Flow cytometry was used to analyze the percentages of TAMs-M1 (M1 macrophages) and TAMs-M2 (M2 macrophages). (**g**) Flow cytometry was used to analyze the percentage of PD-L1 in CD45^-^ cells. (**h**) The percentage of T cells expressing PD-1 in the tumor was analyzed by flow cytometry. * *p* < 0.05, ** *p* < 0.01, *** *p* < 0.001, **** *p* < 0.0001, and ns: no significance.

To further investigate the effect of dual therapy on antitumor immunity, other immune cells in the tumor were examined. The natural killer cells (NKs) (Figure 5a), dendritic cells (DCs) (Figure 5b) and M1 macrophages (Figure 5e) in tumors, which enhance antitumor immune response, were significantly increased in dual-therapy group. However, MDSCs (Figure 5c), Tregs (Figure 5d), and M2 macrophages (Figure 5f), which have an immunosuppressive function in the tumor microenvironment (TME), were also significantly increased in the dual-therapy group. In addition, combination treatment was able to significantly increase the expression of PD-L1 in tumor cells (Figure 5g). Meanwhile, the number of T cells expressing PD-1 increased (Figure 5h).

### 2.6. Anti-PD1 Antibody Further Enhances the Efficacy of Dual Therapy

**Figure 6:**
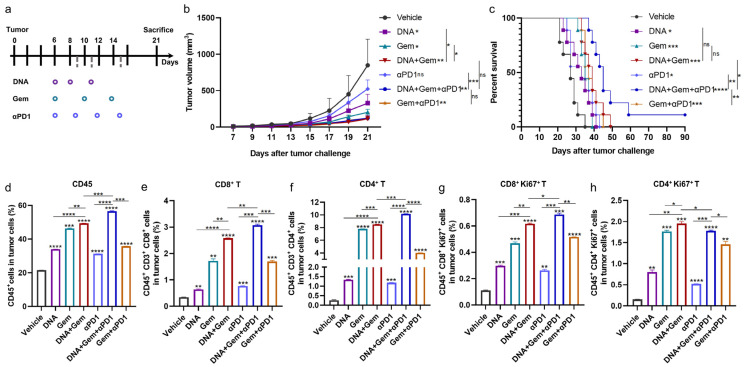
Antitumor effect of triple therapy. (**a**) Schematic immunization schedule, all treatments began 6 days after challenged with tumor cells. (**b**) Tumor growth curves were measured every two days (*n* = 4). (**c**) Survival curves of mice in different treatment groups (*n* = 9). (**d**–**f**) The proportion of infiltrating CD45 (**d**), CD8^+^T cells (**e**), and CD4^+^T cells (**f**) in the tumor was measured by flow cytometry. (**g**,**h**) Percentage of intratumoral proliferative CD8^+^T (CD8^+^Ki67^+^) and proliferative CD4^+^T (CD4^+^Ki67^+^) were analyzed using flow cytometry. * *p* < 0.05, ** *p* < 0.01, *** *p* < 0.001, **** *p* < 0.0001, and ns: no significance.

Considering the increased PD-L1 and PD-1 in tumors after combination therapy, as well as the limited effect of prolonging survival, we continued to explore whether the antitumor efficacy and prolonged survival of dual therapy could be further enhanced by anti-PD1 antibodies. The mice were challenged with MS_f_^+^Lewis cells, and the treatment strategy is shown in Figure 6a. Similar to previous results, the dual therapy significantly enhanced the inhibitory effect on tumor growth compared to single therapy. Compared with the vehicle, anti-PD1 antibody (αPD1) treatment alone inhibited tumor growth but had no significant difference, and similarly, there was no significant difference between the DNA + Gem + αPD1 treatment (triple therapy) and the dual therapy (Figure 6b). However, compared with the dual therapy, the triple therapy significantly extended the survival time of the mice, with one of the mice still free of tumor growth at day 90 (Figure 6c).

The triple therapy was able to further increase the infiltration of intratumoral immune cells (Figure 6d and Figure S4a), with a significant increase in CD8^+^T cells and CD4^+^T cells (Figure 6e,f and Figure S4a). We further analyzed the infiltration of proliferative CD8^+^T cells and CD4^+^T cells, which also showed improvement (Figure 6g,h). In addition, the expression of cytokines in tumors was detected at the mRNA level, and it was found that the expression levels of Gzmb and IFN-γ in the triple therapy were significantly increased (Figure S4b). In summary, the above results indicate that the triple therapy induced more T cell infiltration into tumors and significantly prolonged the survival period of tumor-bearing mice.

### 2.7. The Effect of Triple Therapy on the Tumor Microenvironment (TME)

**Figure 7:**
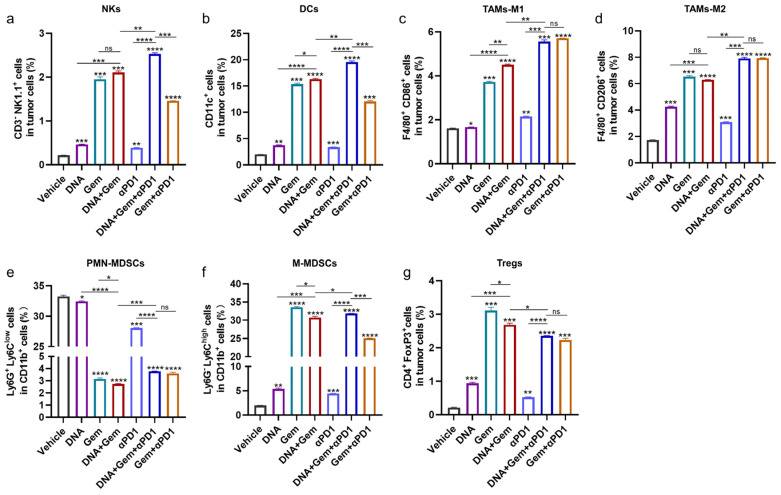
The impact of triple therapy on TME. (**a**,**b**) The percentage of NKs and DCs was analyzed by flow cytometry. (**c**,**d**) Flow cytometry was used to analyze the percentages of M1 macrophages and M2 macrophages. (**e**,**f**) Two types of MDSCs were analyzed by flow cytometry. (**g**) The percentage of Tregs was analyzed by flow cytometry. * *p* < 0.05, ** *p* < 0.01, *** *p* < 0.001, **** *p* < 0.0001, and ns: no significance.

Further analysis of immune cells in the tumor showed that the number of NKs (Figure 7a), DCs (Figure 7b), and M1 macrophages (Figure 7c), which are beneficial for antitumor immune response, were further increased in the triple therapy. However, the numbers of M2 macrophages (Figure 7d), polynuclear MDSCs (PMN-MDSCs) (Figure 7e), and mononuclear MDSCs (M-MDSCs) (Figure 7f) were also increased significantly with triple therapy compared with dual therapy. Although the number of Tregs (Figure 7g) in the triple-therapy group was significantly decreased compared with the dual-therapy group, it was still very high. These results indicate that triple therapy has strong antitumor effects, but if other immune modulators are used to reduce the immunosuppressive cells in the TME, the therapeutic effect may be further enhanced.

## 3. Materials and Methods

### 3.1. Mice and Cell Lines

Female *C57BL/6* mice (6–8 weeks old) were purchased from Liaoning Changsheng Biotechnology Co., Ltd. (Shenyang, China) and raised in the animal experiment platform of the College of Life Sciences, Jilin University. All animal experiments were performed in accordance with Chinese law and were approved by the Ethics Committee of Jilin University (Approval number: YNPZSY2022060).

The Lewis (GF123) cell line was purchased from Shanghai Gefan Biotechnology Co., Ltd., Shanghai, China. MS_f_^+^ Lewis cell lines stably expressing MUC1 and survivin were generated in our lab [34]. Briefly, a GFP-MS plasmid expressing MUC1, survivin, GFP, and G418 resistance genes was constructed and transfected into Lewis cells.

### 3.2. Vaccine Construction

The MS plasmids (expressing a fusion gene of human 9 *MUC1 VNTRs* and human truncated *survivin*-S8, and human *IL-2* is linked by a 2A peptide as a molecular adjuvant) were constructed previously in our laboratory. Human XCL1 was synthesized by the Nanjing Jinsrui Biotechnology Co., Ltd. (Nanjing, China). The plasmid XCL1-MS was constructed using the Seamless Assembly Cloning Kit through homologous recombination following the manufacturer’s protocol and was cloned into the Pst I/BamH I sites of the VR1012 vector. All plasmids contained a Kozak sequence and a tPA signal sequence at the N-terminus of the antigen fragment.

In order to purify the protein, a target gene with His tag was constructed onto the prokaryotic expression vector pET28a. For in vivo targeting, an XCL1-EGFP plasmid was constructed.

### 3.3. Protein Purification

The correct plasmid constructed on the prokaryotic expression vector pET28a was transformed into BL21 (DE3) expression receptor cells. The amplified bacterial solution was transferred to LB medium containing kanamycin and cultured at 220 rpm at 37 °C until the bacterial solution OD value was 0.6–1.0; then, IPTG was added, and the final concentration was 0.5 μM. Protein expression was induced by culture at 180 rpm and 16 °C. After 16–18 h, the bacteria were collected by centrifugation and placed in an ice water bath for ultrasound, the supernatant was collected and filtered by a 0.45 μm filter membrane, the protein was purified by a Ni column, and the target protein was obtained by gradient elution with different concentrations of imidazole.

### 3.4. In Vitro Functional Verification

For T cell activation verification, naïve mouse spleens were isolated and treated in single-cell suspensions. cDC1 were obtained using a CD8^+^ Dendritic Cell Isolation Kit (Miltenyi Biotec) and placed into a 24-well plate at a density of 1 × 10^5^/well. An amount of 0.1 μg MS or XCL1-MS protein was added into the well (two wells per protein), and LPS (100 ng/mL) was added to stimulate cDC1 maturation. The culture was kept for 5 h in an incubator containing 5% CO_2_ at 37 °C. After culture, the polypeptide:MHC-I complex was fixed on the DC surface by adding 0.05% PFA. Then, 2 × 10^5^ CD8^+^ T cells were added to each well, which were sorted using CD8a (Ly-2) MicroBeads (Miltenyi Biotec, Bergisch Gladbach, Germany). An additional 0.5 μg/mL anti-mouse CD28 antibody was added to provide a second signal for CD8^+^T cell activation. After incubation in a 37 °C incubator containing 5% CO_2_ for 24 h, the cells and culture supernatant were collected to analyze the activation of CD8^+^ T cells. The CD69 on the surface of CD8^+^ T cells was analyzed by flow cytometry as the activation marker, and the IFN-γ content secreted by CD8^+^ T cells in the cell culture supernatant was detected by the Mouse IFN-γ ELISA Kit (Absin (Shanghai) Biotechnology Co., Ltd., Shanghai, China) according to the instructions provided by the manufacturer.

For the receptor-binding experiment, 293T cells were transfected with a mouse chemokine receptor XCR1 plasmid. The cells with a good growth state were uniformly spread into a six-well plate and transfected when the cell confluence reached 70~80%. The 2 μg plasmid and 6 μL of Lipofectamine^®^ 2000 DNA Transfection Reagent (Invitrogen, Carlsbad, CA, USA) were diluted using serum-free medium, respectively, and the diluted DNA was added to the diluted transfection reagent (1:1 volume ratio). Incubated at room temperature for 15 min, the DNA-liposome complex was added to the cells. Then, 24 h after transfection, 2 μg of MS or XCL1-MS protein was added to each well, and the binding between the protein and the XCR1 receptor was detected by flow cytometry using the protein’s His tag 48 h after transfection. The His tag was detected using PE anti-His Tag Antibody (RRID: AB_2563634) from BioLegend (San Diego, CA, USA). The receptor-binding experiments were performed twice with similar trends.

### 3.5. In Vivo Immune Strategies

For immunogenicity analysis, the vaccine plasmid or vector was intramuscularly injected into the tibialis anterior muscles of both hind limbs (50 μg in each limb) of *C57BL/6* mice on days 0, 2, and 5. One week after the last immunization, the mice were euthanized.

For antitumor effect analysis, 5 × 10^5^ MS_f_^+^ Lewis cells were injected subcutaneously into the right hind flank of the *C57BL/6* mice on day 0. The vaccine plasmid or vector was intramuscularly injected into the anterior tibial muscle (50 μg in each limb) on days 6, 8, and 11. To investigate the antitumor effect of the vaccine combined with gemcitabine, mice were given gemcitabine (50 mg/kg, intraperitoneally injected) on days 6, 10, and 14. For anti-PD1 antibodies, these were intraperitoneally injected at a dose of 100 μg/mouse on days 6, 9, 12, and 15. The anti-PD1 antibody used in vivo was derived from BioXcell (Lebanon, PA, USA) (catalogue number: BE0146; RRID: AB_10949053). The tumor size was measured every two days using a vernier caliper and the tumor volume was calculated by (length × width^2^)/2. For survival studies, the weight and tumor volume of tumor-bearing mice was monitored, and the mice were euthanized when they reached the humane endpoint (including the tumor size reaching 1500 mm^3^) or had severe weight loss.

### 3.6. ELISpot and Cytotoxicity Assays

IFN-γ ELISpot assays were performed using the ELISpot kit (BD Biosciences, Franklin Lakes, NJ, USA) according to the instructions provided by the manufacturer, and in vitro cytotoxicity assays were performed based on previous studies [33]. Simply put, Lewis cells were used as target cells and labeled with antigen peptides (final concentration: 5 μg/mL) or without antigen peptides and incubated for 2 h. Target cells incubated with peptides were stained with high concentrations (5 μM) of CFSE, while non-peptide-labeled target cells were stained with low concentrations (0.5 μM) of CFSE. Target cells stained with high and low concentrations of CFSE were evenly mixed 1:1 (a total of 1 × 10^5^ target cells), and then different numbers of spleen cells (effector cells) from vaccinated mice were mixed with target cells in different ratios. Effector cells and target cells were incubated at 37 °C for 8 h, and cytotoxicity was analyzed by flow cytometry, which detects a decrease in the percentage of specific target cells.

### 3.7. Cell Staining and Flow Cytometry

For mouse tumor cell staining, tumor tissue was removed from the mice, then cut and digested with liberase (Roche, Basel, Switzerland) and DNase I (Sigma, Burlington, MA, USA) for 2 h. After washing and counting the cells, extracellular staining was performed with fluorescently labeled antibodies. A total of 2 × 10^6^ cells were suspended in 200 µL of cell-staining buffer, anti-mouse CD16/32 Antibody (AB_1574973) was added, and then it was incubated on ice for 5–10 min for blocking. The antibodies were then added and incubated on ice in the dark for 15 min.

For intracellular staining, after completing antibody staining on the cell surface as mentioned above, 1 mL of fixing buffer (eBioscience, San Diego, CA, USA) was added and placed away from light for 1 h to fix the cells. It was centrifuged at 350× *g* for 5 min; the supernatant was discarded and washed twice with Permeabilization Buffer (eBioscience). The fixed and permeated cells were re-suspended in the cell-staining buffer, added to antibodies, and incubated at room temperature in the dark for 30 min.

The main antibodies used in the experiment were as follows: FITC anti-mouse CD3ε Antibody (AB_312671); APC anti-mouse CD8a Antibody (AB_312751); PE/Cyanine7 anti-mouse CD4 Antibody (AB_312729); PE anti-His Tag Antibody (AB_2563634); APC anti-mouse XCR1 Antibody (AB_2563931); PE anti-mouse CD69 Antibody (AB_313110); PE anti-mouse Ki-67 Antibody (AB_2716014); PE/Cyanine7 anti-mouse CD45 Antibody (AB_312979). All antibodies used in the staining were from Biolegend. After staining with antibodies and washing twice by centrifugation at 350× *g* for 5 min, all samples were analyzed by flow cytometry.

### 3.8. Quantitative Real-Time PCR (qRT-PCR)

The total RNA of tumor samples was isolated with Trizol reagent (Invitrogen), and mRNA was reverse-transcribed to cDNA using the PrimeScript 1st Strand cDNA Synthesis kit (Takara, San Jose, CA, USA). qRT-PCR was used to quantify mRNA levels. The primers and qRT-PCR program used to detect Gzmb, IFN-γ, IL-2, and TNFα mRNA expression were described previously [39].

### 3.9. Statistical Analysis

The data were analyzed using an unpaired *t*-test or two-way ANOVA, and the results were expressed as mean ± standard error. The statistical analysis of survival data was performed using a log-rank test. All statistical analyses were performed in GraphPad Prism 8.0. * *p* < 0.05, ** *p* < 0.01, *** *p* < 0.001, **** *p* < 0.0001, and ns: no significance.

## 4. Discussion

cDC1 play a pivotal role in cross-presenting antigens to activate CD8^+^T cells, a process vital for enhancing the immunogenicity and antitumor functionality of DNA vaccines [40,41]. Consequently, targeting cDC1 emerges is a promising strategy in vaccine design. Previous research focused primarily on DEC-205 and Clec9A, demonstrating their ability to augment the immune response and inhibit tumor growth [42,43,44]. However, their expression on the surface of cDC1 lacks specificity [10]. In this study, we specifically targeted XCR1, expressed on the surface of cDC1, and the DNA vaccine that fused its ligand-XCL1 with two tumor-associated antigens showed stronger immunogenicity and antitumor effects in mice.

cDC1 is particularly good at transporting tumor antigens to tumor-draining lymph nodes to initiate the antitumor CD8^+^T cell response [17,45]. Therefore, the abundance of cDC1 in tumors is crucial for the antitumor immune response. Research has shown that NKs can produce chemokines CCL5 and XCL1 to recruit cDC1 into the tumor microenvironment, which is necessary for tumor immune control, and the disruption of this process can lead to cancer immune escape [46]. This suggests that the intratumoral delivery of XCL1 appears to be a promising strategy for enhancing cDC1 recruitment [47]. In addition, the recruitment of cDC1 within tumors has other benefits, including the production of chemokines CXCL9 and CXCL10 to recruit CXCR3 effector T cells for tumor infiltration, and a further enhancement of the antitumor activity of T cells and NKs by cytokine IL-12 [12,48].

However, during the progression of cancer, the tumor microenvironment can induce the production of a large number of immunosuppressive cells or factors, hindering effective antitumor immune responses [49]. It is worth noting that in the lung cancer mouse model, the use of the chemotherapy drug Gem improves the therapeutic effect while upregulating a variety of immunosuppressive cells. Although, there have been reports in other tumor models that Gem can help downregulate MDSC levels in the tumor microenvironment [37]. However, MDSCs were composed of two subgroups; our results show that Gem application resulted in a significant decrease in the number of PMN-MDSCs and a significant increase in the number of M-MDSCs. This is consistent with previous reports that the tumor microenvironment after gemcitabine treatment is favorable for M-MDSC differentiation [50]. In addition, Gem upregulates the expression of PD-L1 and PD-1, which is consistent with the literature reports in [37,39]. Gem also resulted in a significant increase in the number of Tregs. This suggests that we need to find a suitable immunomodulator to remove immune suppression in the tumor microenvironment to further improve the effect of cancer immunotherapy.

## Data Availability

The datasets used and/or analysis during the current study are available from the corresponding author on reasonable request.

## Supplementary Materials

The following supporting information can be downloaded at: https://www.mdpi.com/article/10.3390/ijms25031880/s1.

## References

[1] Yang L., Ning Q., Tang S.-S. (2022). Recent Advances and Next Breakthrough in Immunotherapy for Cancer Treatment. J. Immunol. Res..

[2] Schaft N., Dörrie J., Schuler G., Schuler-Thurner B., Sallam H., Klein S., Eisenberg G., Frankenburg S., Lotem M., Khatib A. (2023). The future of affordable cancer immunotherapy. Front. Immunol..

[3] Iwahori K., Birbrair A. (2020). Cytotoxic CD8+ Lymphocytes in the Tumor Microenvironment. Tumor Microenvironment: Hematopoietic Cells—Part A.

[4] Ott P.A., Wu C.J. (2019). Cancer Vaccines: Steering T Cells Down the Right Path to Eradicate Tumors. Cancer Discov..

[5] Saxena M., van der Burg S.H., Melief C.J.M., Bhardwaj N. (2021). Therapeutic cancer vaccines. Nat. Rev. Cancer.

[6] Lopes A., Vandermeulen G., Préat V. (2019). Cancer DNA vaccines: Current preclinical and clinical developments and future perspectives. J. Exp. Clin. Cancer Res..

[7] Hu Z., Ott P.A., Wu C.J. (2018). Towards personalized, tumour-specific, therapeutic vaccines for cancer. Nat. Rev. Immunol..

[8] Santos P.M., Butterfield L.H. (2018). Dendritic Cell-Based Cancer Vaccines. J. Immunol..

[9] Fu C., Ma T., Zhou L., Mi Q.-S., Jiang A. (2022). Dendritic Cell-Based Vaccines Against Cancer: Challenges, Advances and Future Opportunities. Immunol. Investig..

[10] Matsuo K., Yoshie O., Kitahata K., Kamei M., Hara Y., Nakayama T. (2021). Recent Progress in Dendritic Cell-Based Cancer Immunotherapy. Cancers.

[11] Eisenbarth S.C. (2019). Dendritic cell subsets in T cell programming: Location dictates function. Nat. Rev. Immunol..

[12] Böttcher J.P., Reis e Sousa C. (2018). The Role of Type 1 Conventional Dendritic Cells in Cancer Immunity. Trends Cancer.

[13] den Haan J.M.M., Lehar S.M., Bevan M.J. (2000). Cd8+ but Not Cd8− Dendritic Cells Cross-Prime Cytotoxic T Cells in Vivo. J. Exp. Med..

[14] Pooley J.L., Heath W.R., Shortman K. (2001). Cutting Edge: Intravenous Soluble Antigen Is Presented to CD4 T Cells by CD8− Dendritic Cells, but Cross-Presented to CD8 T Cells by CD8+ Dendritic Cells1. J. Immunol..

[15] Audsley K.M., McDonnell A.M., Waithman J. (2020). Cross-Presenting XCR1+ Dendritic Cells as Targets for Cancer Immunotherapy. Cells.

[16] Meyer M.A., Baer J.M., Knolhoff B.L., Nywening T.M., Panni R.Z., Su X., Weilbaecher K.N., Hawkins W.G., Ma C., Fields R.C. (2018). Breast and pancreatic cancer interrupt IRF8-dependent dendritic cell development to overcome immune surveillance. Nat. Commun..

[17] Salmon H., Idoyaga J., Rahman A., Leboeuf M., Remark R., Jordan S., Casanova-Acebes M., Khudoynazarova M., Agudo J., Tung N. (2016). Expansion and Activation of CD103(+) Dendritic Cell Progenitors at the Tumor Site Enhances Tumor Responses to Therapeutic PD-L1 and BRAF Inhibition. Immunity.

[18] Spranger S., Dai D., Horton B., Gajewski T.F. (2017). Tumor-Residing Batf3 Dendritic Cells Are Required for Effector T Cell Trafficking and Adoptive T Cell Therapy. Cancer Cell.

[19] Bachem A., Hartung E., Güttler S., Mora A., Zhou X., Hegemann A., Plantinga M., Mazzini E., Stoitzner P., Gurka S. (2012). Expression of XCR1 Characterizes the Batf3-Dependent Lineage of Dendritic Cells Capable of Antigen Cross-Presentation. Front. Immunol..

[20] Dorner B.G., Dorner M.B., Zhou X., Opitz C., Mora A., Güttler S., Hutloff A., Mages H.W., Ranke K., Schaefer M. (2009). Selective expression of the chemokine receptor XCR1 on cross-presenting dendritic cells determines cooperation with CD8+ T cells. Immunity.

[21] Crozat K., Guiton R., Contreras V., Feuillet V., Dutertre C.A., Ventre E., Vu Manh T.P., Baranek T., Storset A.K., Marvel J. (2010). The XC chemokine receptor 1 is a conserved selective marker of mammalian cells homologous to mouse CD8alpha+ dendritic cells. J. Exp. Med..

[22] Bachem A., Güttler S., Hartung E., Ebstein F., Schaefer M., Tannert A., Salama A., Movassaghi K., Opitz C., Mages H.W. (2010). Superior antigen cross-presentation and XCR1 expression define human CD11c+CD141+ cells as homologues of mouse CD8+ dendritic cells. J. Exp. Med..

[23] Jongbloed S.L., Kassianos A.J., McDonald K.J., Clark G.J., Ju X., Angel C.E., Chen C.J., Dunbar P.R., Wadley R.B., Jeet V. (2010). Human CD141+ (BDCA-3)+ dendritic cells (DCs) represent a unique myeloid DC subset that cross-presents necrotic cell antigens. J. Exp. Med..

[24] Poulin L.F., Salio M., Griessinger E., Anjos-Afonso F., Craciun L., Chen J.L., Keller A.M., Joffre O., Zelenay S., Nye E. (2010). Characterization of human DNGR-1+ BDCA3+ leukocytes as putative equivalents of mouse CD8alpha+ dendritic cells. J. Exp. Med..

[25] Qi H., Sun Z., Yao Y., Chen L., Su X. (2022). Immunogenicity of the Xcl1-SARS-CoV-2 Spike Fusion DNA Vaccine for COVID-19. Vaccines.

[26] Fossum E., Grødeland G., Terhorst D., Tveita A.A., Vikse E., Mjaaland S., Henri S., Malissen B., Bogen B. (2015). Vaccine molecules targeting Xcr1 on cross-presenting DCs induce protective CD8+ T-cell responses against influenza virus. Eur. J. Immunol..

[27] Chen K., Wu Z., Zhao H., Wang Y., Ge Y., Wang D., Li Z., An C., Liu Y., Wang F. (2020). XCL1/Glypican-3 Fusion Gene Immunization Generates Potent Antitumor Cellular Immunity and Enhances Anti-PD-1 Efficacy. Cancer Immunol. Res..

[28] Le Gall C., Cammarata A., de Haas L., Ramos-Tomillero I., Cuenca-Escalona J., Schouren K., Wijfjes Z., Becker A.M.D., Bödder J., Dölen Y. (2022). Efficient targeting of NY-ESO-1 tumor antigen to human cDC1s by lymphotactin results in cross-presentation and antigen-specific T cell expansion. J. Immunother. Cancer.

[29] Hartung E., Becker M., Bachem A., Reeg N., Jäkel A., Hutloff A., Weber H., Weise C., Giesecke C., Henn V. (2015). Induction of Potent CD8 T Cell Cytotoxicity by Specific Targeting of Antigen to Cross-Presenting Dendritic Cells In Vivo via Murine or Human XCR1. J. Immunol..

[30] Gao T., Cen Q., Lei H. (2020). A review on development of MUC1-based cancer vaccine. Biomed. Pharmacother. Biomed. Pharmacother..

[31] Garg H., Suri P., Gupta J.C., Talwar G.P., Dubey S. (2016). Survivin: A unique target for tumor therapy. Cancer Cell Int..

[32] Zhang H., Liu C., Zhang F., Geng F., Xia Q., Lu Z., Xu P., Xie Y., Wu H., Yu B. (2016). MUC1 and survivin combination tumor gene vaccine generates specific immune responses and anti-tumor effects in a murine melanoma model. Vaccine.

[33] Guo Q., Wang L., Xu P., Geng F., Guo J., Dong L., Bao X., Zhou Y., Feng M., Wu J. (2020). Heterologous prime-boost immunization co-targeting dual antigens inhibit tumor growth and relapse. Oncoimmunology.

[34] Liu C., Lu Z., Xie Y., Guo Q., Geng F., Sun B., Wu H., Yu B., Wu J., Zhang H. (2018). Soluble PD-1-based vaccine targeting MUC1 VNTR and survivin improves anti-tumor effect. Immunol. Lett..

[35] Kerr M.D., McBride D.A., Chumber A.K., Shah N.J. (2021). Combining therapeutic vaccines with chemo- and immunotherapies in the treatment of cancer. Expert. Opin. Drug Discov..

[36] Wang H., He X., Fang D., Wang X., Guan J., Shi Z.W., Chen X. (2022). Gemcitabine-facilitated modulation of the tumor microenvironment and PD-1/PD-L1 blockade generate a synergistic antitumor effect in a murine hepatocellular carcinoma model. Clin. Res. Hepatol. Gastroenterol..

[37] Tomihara K., Fuse H., Heshiki W., Takei R., Zhang B., Arai N., Nakamori K., Noguchi M. (2014). Gemcitabine chemotherapy induces phenotypic alterations of tumor cells that facilitate antitumor T cell responses in a mouse model of oral cancer. Oral Oncol..

[38] Du B., Wen X., Wang Y., Lin M., Lai J. (2020). Gemcitabine and checkpoint blockade exhibit synergistic anti-tumor effects in a model of murine lung carcinoma. Int. Immunopharmacol..

[39] Geng F., Dong L., Bao X., Guo Q., Guo J., Zhou Y., Yu B., Wu H., Wu J., Zhang H. (2022). CAFs/tumor cells co-targeting DNA vaccine in combination with low-dose gemcitabine for the treatment of Panc02 murine pancreatic cancer. Mol. Ther. Oncolytics.

[40] Macri C., Jenika D., Ouslinis C., Mintern J.D. (2023). Targeting dendritic cells to advance cross-presentation and vaccination outcomes. Semin. Immunol..

[41] Baljon J.J., Wilson J.T. (2022). Bioinspired vaccines to enhance MHC class-I antigen cross-presentation. Curr. Opin. Immunol..

[42] Macri C., Dumont C., Johnston A.P., Mintern J.D. (2016). Targeting dendritic cells: A promising strategy to improve vaccine effectiveness. Clin. Transl. Immunol..

[43] Cao J., Jin Y., Li W., Zhang B., He Y., Liu H., Xia N., Wei H., Yan J. (2013). DNA vaccines targeting the encoded antigens to dendritic cells induce potent antitumor immunity in mice. BMC Immunol..

[44] Sancho D., Mourão-Sá D., Joffre O.P., Schulz O., Rogers N.C., Pennington D.J., Carlyle J.R., Reis e Sousa C. (2008). Tumor therapy in mice via antigen targeting to a novel, DC-restricted C-type lectin. J. Clin. Investig..

[45] Roberts E.W., Broz M.L., Binnewies M., Headley M.B., Nelson A.E., Wolf D.M., Kaisho T., Bogunovic D., Bhardwaj N., Krummel M.F. (2016). Critical Role for CD103(+)/CD141(+) Dendritic Cells Bearing CCR7 for Tumor Antigen Trafficking and Priming of T Cell Immunity in Melanoma. Cancer Cell.

[46] Böttcher J.P., Bonavita E., Chakravarty P., Blees H., Cabeza-Cabrerizo M., Sammicheli S., Rogers N.C., Sahai E., Zelenay S., Reis e Sousa C. (2018). NK Cells Stimulate Recruitment of cDC1 into the Tumor Microenvironment Promoting Cancer Immune Control. Cell.

[47] Cancel J.C., Crozat K., Dalod M., Mattiuz R. (2019). Are Conventional Type 1 Dendritic Cells Critical for Protective Antitumor Immunity and How?. Front. Immunol..

[48] Bergamaschi C., Pandit H., Nagy B.A., Stellas D., Jensen S.M., Bear J., Cam M., Valentin A., Fox B.A., Felber B.K. (2020). Heterodimeric IL-15 delays tumor growth and promotes intratumoral CTL and dendritic cell accumulation by a cytokine network involving XCL1, IFN-γ, CXCL9 and CXCL10. J. Immunother. Cancer.

[49] Pitt J.M., Marabelle A., Eggermont A., Soria J.C., Kroemer G., Zitvogel L. (2016). Targeting the tumor microenvironment: Removing obstruction to anticancer immune responses and immunotherapy. Ann. Oncol. Off. J. Eur. Soc. Med. Oncol..

[50] Wu C., Tan X., Hu X., Zhou M., Yan J., Ding C. (2020). Tumor Microenvironment following Gemcitabine Treatment Favors Differentiation of Immunosuppressive Ly6C(high) Myeloid Cells. J. Immunol..

