# Supplementary material for: The XCL1-Mediated DNA Vaccine Targeting Type 1 Conventional Dendritic Cells Combined with Gemcitabine and Anti-PD1 Antibody Induces Potent Antitumor Immunity in a Mouse Lung Cancer Model
Source: Int J Mol Sci. 2024 Feb 4;25(3):1880. doi: 10.3390/ijms25031880 (PMC10855623; doi:10.3390/ijms25031880)
Supplement: Supplementary file 1 [file ijms-25-01880-s001.zip › ijms-2784199-supplementary.pdf]

Supplement Figure

Figure S1

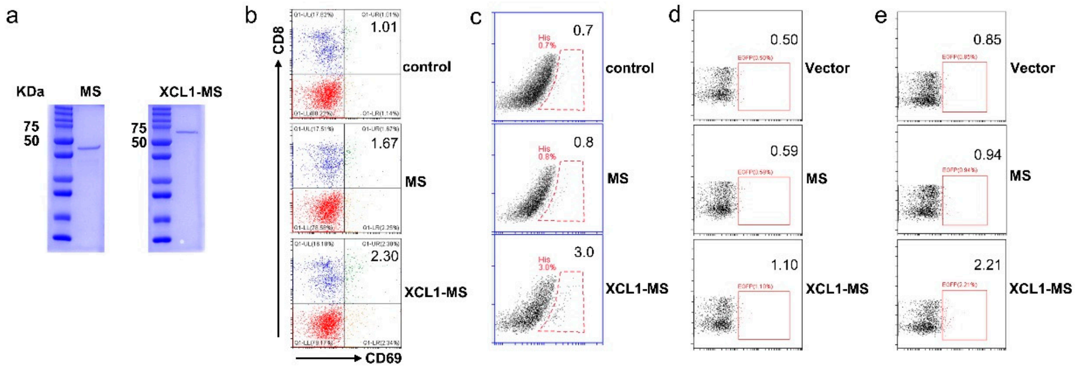

Figure S2

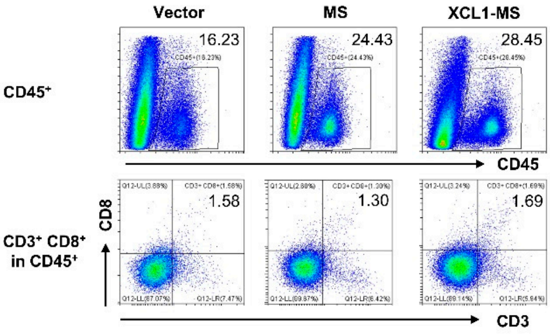

Figure S3

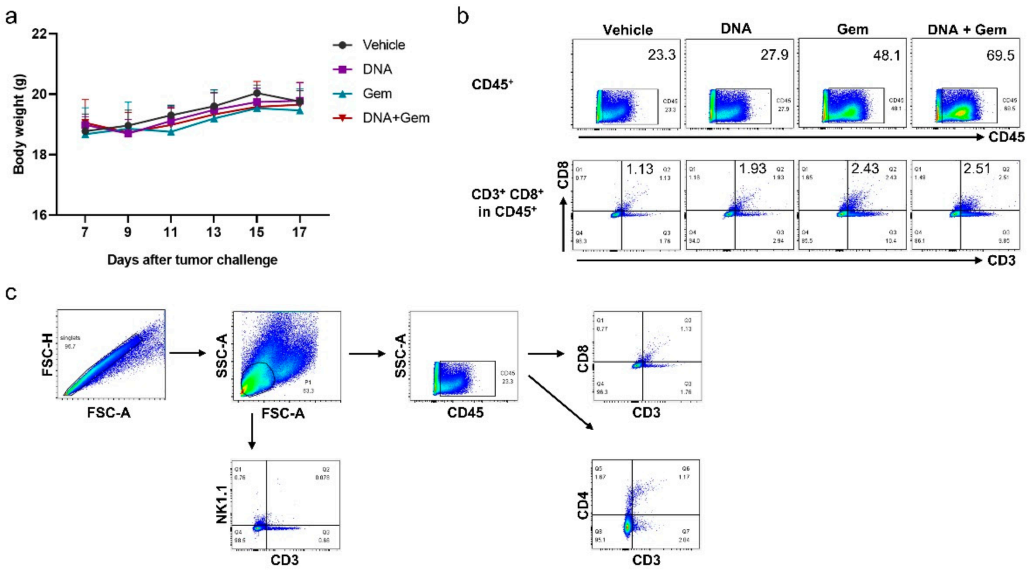

Figure S4

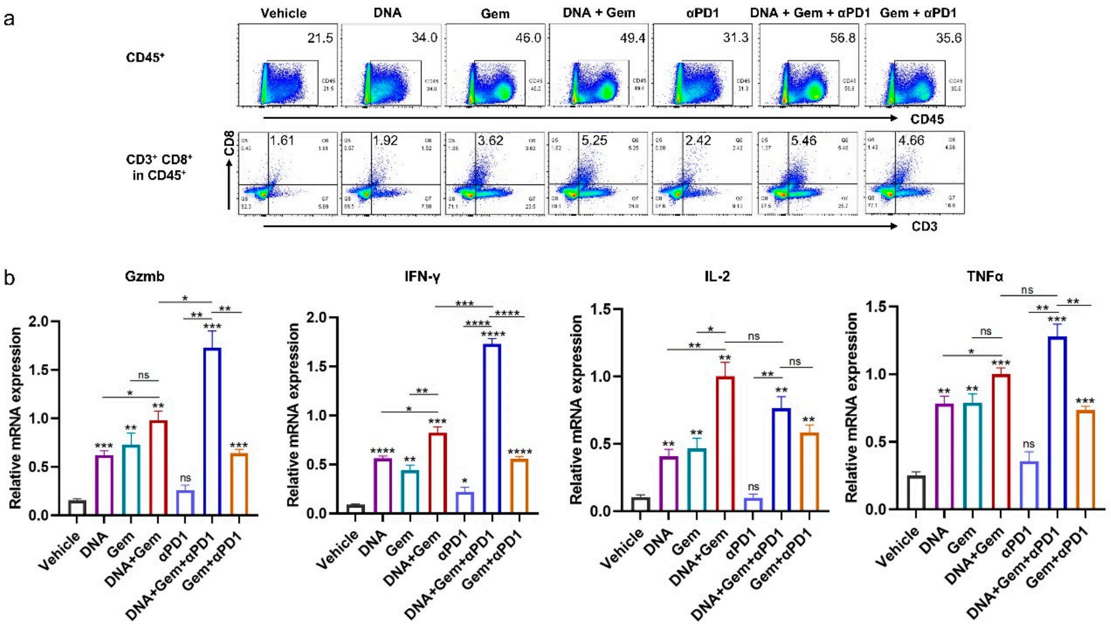

Figure Captions

Figure S1: Representative Flow Cytometry Images. (a) The proteins MS (left) and XCL1-MS (right) were purified for functional validation in vitro. (b) Flow cytometry analysis of $CD8^+$ T cell activation as shown in Figure 1c. (c) Flow cytometry analysis of receptor binding, as presented in Figure 1e. (d) Flow cytometry analysis of cDC1 targeting three days post-immunization, as depicted in Figure 1f. (e) Flow cytometry analysis of cDC1 targeting six days post-immunization, as illustrated in Figure 1g.

Figure S2: Representative Images of Infiltrating Immune Cells in Tumors Using Flow Cytometry. These images represent the proportion of CD45 and $CD8^+$ T cells in tumors following DNA vaccine immunization.

Figure S3: Effect of DNA and Gem Combined Therapy on Body Weight and Infiltrating Immune Cells in Tumors. (a) Gem administration led to slight weight loss, with no significant difference compared to other groups. (b) These images represent the proportion of CD45 and $CD8^+$ T cells in tumors after immunization with different therapies. (c) Representative gating strategies for flow cytometry experiments. The gating strategy of other cells in the tumor is the same as the NKs strategy in the figure, and different markers are shown as the ordinates in each figure.

Figure S4: Flow Cytometry Images of Infiltrating Immune Cells in Tumors and qRT-PCR Analysis. (a) These images represent the proportion of CD45 and $CD8^+$ T cells in tumors after immunization with different therapies. (b) qRT-PCR analysis of relative mRNA expression levels of Gzmb, IFN-γ, IL-2, and TNF in mouse tumors. GAPDH served as an endogenous control. Statistical significance: *$P < 0.05$, **$P < 0.01$, ***$P < 0.001$, ****$P < 0.0001$, ns, no significance.
